# SPAN 60 and TWEEN 60 surfactant-based niosomal vesicles: from preparation to molecular dynamics simulations

**DOI:** 10.1039/d6na00065g

**Published:** 2026-08-03

**Authors:** Martina Ježková, Ashley H. George, Mario Vazdar, Jiří Mikšátko, Jaroslav Hanuš, Miroslav Šoós

**Affiliations:** a Department of Chemical Engineering, University of Chemistry and Technology Prague Technická 5 166 28 Prague 6 Czech Republic miroslav.soos@vscht.cz; b Imaging Methods Core Facility, Charles University in Prague, Faculty of Science Prague Czech Republic; c Department of Mathematics, Informatics, and Cybernetics, University of Chemistry and Technology Prague Technická 5 166 28 Prague 6 Czech Republic

## Abstract

The role of vesicular systems in the field of drug delivery has led to increased research in the optimization of these novel delivery methods. Niosomes are bilayer structures that are capable of encapsulating lipophilic and hydrophilic compounds. In this study, niosomal systems formed from either Span 60 or Tween 60 non-ionic surfactants were investigated through experimental work and molecular dynamics simulations. Niosomes were prepared using thin-film hydration (TFH) and organic phase injection (OPI) methods and characterized in terms of particle size, polydispersity index, zeta potential, morphology, and encapsulation efficiency. A low-molecular-weight active pharmaceutical ingredient, tetracaine, and its water-soluble form, tetracaine HCl, were employed to understand the effect of pH on the encapsulation efficiency (EE) of the process. The molecular dynamics simulation of the niosomal system provided information on the distribution of Span 60 or Tween 60 surfactant molecules within the bilayer and the behavior of the tetracaine molecule.

## Abstract 2

Exploring tetracaine encapsulation in Span 60 and Tween 60 niosomes through experimental characterization and molecular dynamics simulations.

## Graphical abstract

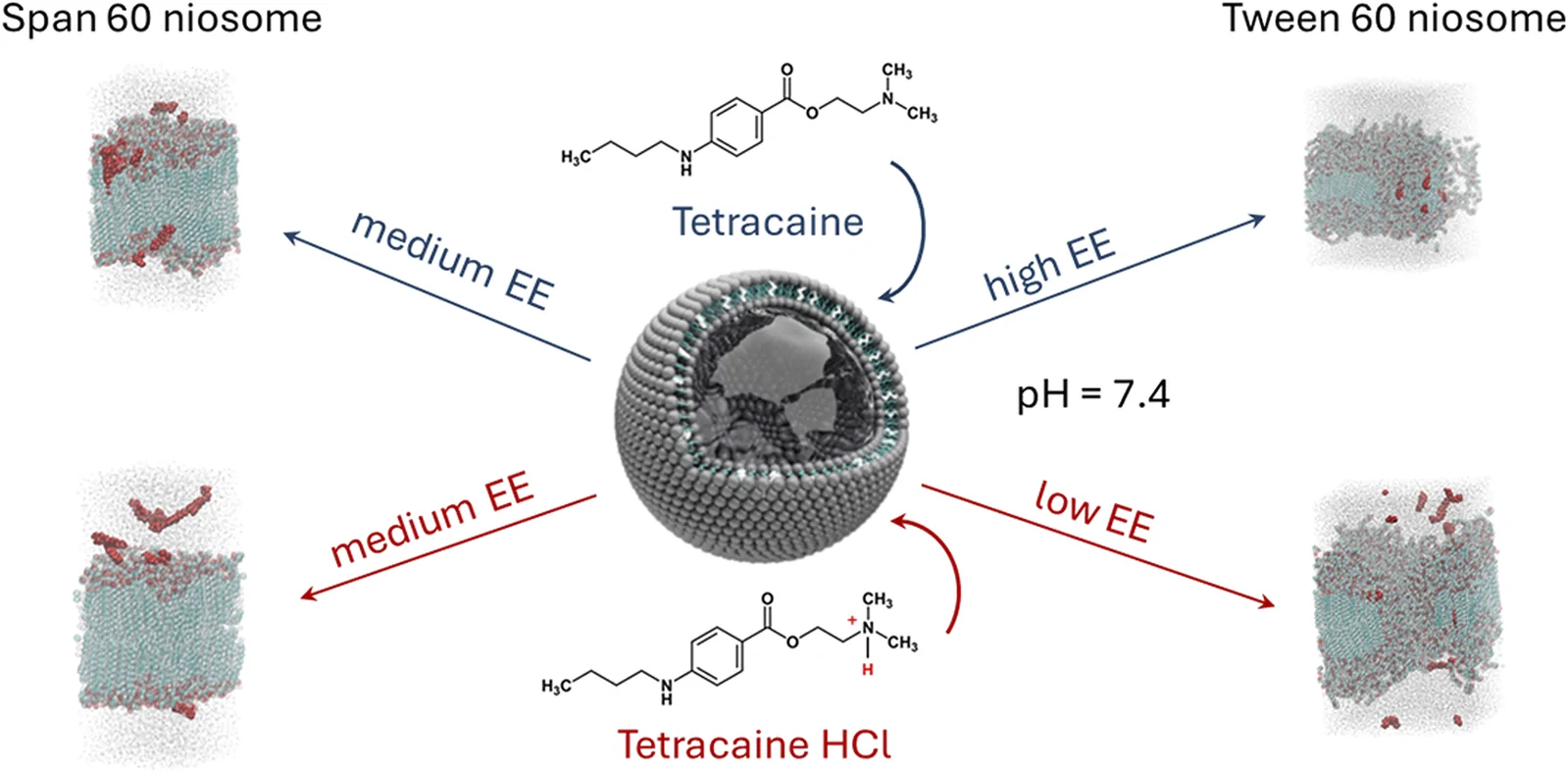

## 1 Introduction

Niosomes are vesicular carriers, typically ranging from approximately 10 nm to 100 µm in diameter, composed of a bilayer membrane surrounding an aqueous core.^1,2^ Their membrane is made up of nonionic surfactants with optionally added supporting agents that influence the niosomal shell stability, rigidity, or the protection of a loaded cargo.^3,4^ Niosomes were developed by the L'Oréal company for cosmetic and personal care products in the 1970s^5^ and patented in the 1980s.^6^ For the first time, the niosomal formulation was introduced to the general public in the treatment cream called Niosome produced by Lancôme in 1986.^7^ The L'Oréal company synthesized alkyl glycerol ethers for the preparation of niosomes to overcome the disadvantages of phospholipid-based liposomes.^5,8,9^ In recent years, niosomal systems have gained renewed attention due to advances in formulation strategies, characterization techniques, and expanded biomedical applications. While originally developed for cosmetic use, the research has increasingly shifted toward pharmaceutical and therapeutic applications, including transdermal, ocular, and targeted drug delivery systems.^10–13^ Studies show that niosomes are flexible drug delivery systems that can improve the solubility, stability, and bioavailability of active compounds.^14–17^

Although nonionic surfactants are not charged, they comprise the hydrophilic and hydrophobic parts that interact *via* hydrogen bonds. Ethylene oxide and multihydroxy-based surfactants are two main groups of nonionic surfactants, whose main building units can also be combined.^18^ In addition to alkyl glycerol ethers synthesized particularly for the topical applications in the cosmetic industry,^5^ other molecules acting as nonionic surfactants were reported to be useful for the preparation of niosomes, including alkyl glycosides, alkyl esters, alkyl amides, fatty acids, or amino acids.^19^ Nowadays, commercially produced surfactants are often employed for the preparation of niosomal dispersions, such as Tween, Span, or Brij families.^20–22^

The surfactant affinity to polar and non-polar phases is described by the HLB.^23,24^ In general, surfactants with low HLB values (typically below approximately 8) are considered more lipophilic, whereas surfactants with high HLB values (typically above approximately 10) are more hydrophilic. The HLB value influences niosome formation, stability, permeability, and drug retention. Surfactants with higher HLB values tend to promote interactions with aqueous environments and facilitate membrane permeability, whereas lower HLB surfactants typically form more rigid and less permeable bilayers, which can improve retention of encapsulated compounds.^25^ Thus, a drug formulation that uses niosomes can be tailored by selecting suitable building blocks. Size distribution, chemical composition, shelf-life stability, or sufficient resistance to external stress are some of the important factors that influence the formulation of niosomal drug carriers.

Niosomes have been reported to encapsulate a wide range of bioactive compounds, including both hydrophilic and lipophilic molecules.^26–30^ Although drug release profiles are less frequently analyzed and mentioned in the literature, available data indicate the niosomal capability to effectively entrap model compounds.^19,31^ However, encapsulation efficiency (EE) strongly depends on factors such as surfactant type, membrane composition, preparation method, and drug physicochemical properties. While high EE values have been reported in optimized systems, they are not universally achieved, and variability remains a key challenge. Despite their advantages, niosomal drug delivery systems may suffer from formulation-dependent instability, leakage of encapsulated compounds, and sensitivity to processing conditions. In addition to the essential niosomal components, the addition of supporting agents, such as cholesterol,^32^ dicethyl phosphate, *N*-cetylpyridinium chloride,^27^ and fatty alcohols,^33^ or the freezing, thawing and extrusion treatment^8,34,35^ can be used to further improve the properties of produced niosomal formulations. Furthermore, the effect of pH on the encapsulation of ionizable compounds in niosomal systems has not been fully resolved, especially in relation to surfactant type and preparation method. The ionization state of drugs can significantly alter their affinity toward the aqueous phase or the membrane, thereby affecting encapsulation efficiency and localization within the vesicle. However, a systematic investigation combining experimental data with molecular-level insight is still lacking.

In this work, we address these gaps by comparing niosomes prepared from Span 60 and Tween 60 as representative low-HLB and high-HLB surfactants. Using tetracaine and tetracaine hydrochloride as model compounds, we investigate the influence of surfactant type, drug form, preparation method, and pH on encapsulation efficiency, vesicle size, and structural properties. Experimental results are complemented by all-atom molecular dynamics simulations to provide detailed insight into bilayer organization and drug distribution within the niosomal systems.

## 2 Materials and methods

### 2.1 Chemicals

All chemicals were used as received without further purification. The following chemicals were bought from Sigma-Aldrich (Germany): Span 60, Tween 60, cholesterol (92.5%), tetracaine (98%), and tetracaine hydrochloride (HCl) (99%). The following chemicals were bought from Penta (Czech Republic): diethyl ether (p.a.), ethanol (p.a., 99.5%), methanol (p.a., 99.5%), isopropyl alcohol (99.5%), sodium hydroxide (p.a.) and hydrochloric acid (p.a., 35%). Aqual® Ultrapur demineralized water was used for all experiments. Amicon Ultra 0.5 mL MWCO 3 kDa centrifugal filters were used to separate the aqueous environment containing unloaded drug from niosomes.

### 2.2 The preparation of plain and drug-loaded niosomes

Drug-loaded niosomes were prepared by the hydration of a thin film (TFH method: TFHm)^36^ and by organic phase injection (OPI method: OPIm).^9^ The organic phase consisted of isopropyl alcohol (IPA) for Span 60 formulations and diethyl ether for Tween 60 formulations, in which the surfactant and cholesterol were dissolved. The drug was introduced into either the organic or aqueous phase depending on its solubility; specifically, tetracaine hydrochloride (TCNH) was dissolved in the aqueous phase, while tetracaine (TCN) was dissolved in the organic phase.

In the OPIm method, the organic phase containing the dissolved components was added dropwise (0.2 mL min^−1^) into 40 mL of preheated demineralized water under magnetic stirring. The aqueous phase was preheated to 90 °C for Span 60 systems and 55 °C for Tween 60 systems, which were temperatures above the boiling point of the used solvent. During the process, the organic solvent was allowed to evaporate until a final volume of 40 mL was reached. For the TFHm method, 10 mL of the organic solution was evaporated in a 50 mL round-bottom flask using a rotary evaporator (Rotavapor® R-100, BUCHI, Switzerland) and subsequently dried overnight in a desiccator to form a thin film. The film was then hydrated with demineralized water to a final volume of 40 mL and sonicated for 60 min using a probe sonicator (Hielscher UP400St with sonotrode S24d3, Germany, 80 W, amplitude cycle of 70%), while the flask was cooled in an ice bath.

For all formulations, the molar ratio of the surfactant to cholesterol was 3 : 1 (50 mg of surfactant). In the absence of cholesterol, Tween 60 did not form stable niosomes, whereas Span 60 formed a stable suspension; therefore, cholesterol was included in all formulations for consistency. The final drug concentration in the niosomal suspension was 0.25 mg mL^−1^.

For pH-dependent experiments, the aqueous phase was adjusted to pH 5.3, 6.3, 7.4, 9.3, and 11 by addition of hydrochloric acid or sodium hydroxide prior to niosome preparation.

### 2.3 Freezing–thawing–extrusion treatment applied after the preparation of niosomes

A 13 mL aliquot of the niosomal suspension was transferred into a 15 mL Falcon tube and fully immersed in liquid nitrogen for 5 min (freezing step, F). The sample was then thawed in a water bath at 65 °C for 15 min (thawing step, T). A temperature of 65 °C was selected to ensure sufficient bilayer fluidity during vesicle reorganization, as it is above the phase transition temperature (Tm of around 55–60 °C) of Span 60 and the same temperature was used for Tween 60 to maintain consistent processing conditions.^37^ This freezing–thawing (FT) cycle was repeated 2, 5, or 10 times, which was used to evaluate the effect of repeated bilayer disruption and self-assembly nature on encapsulation efficiency and vesicle size. Following the FT treatment, extrusion was performed using an Avanti Mini Extruder (Avanti Polar Lipids, USA). A 1 mL sample of the niosomal suspension was loaded into a glass syringe and passed through a 400 nm membrane filter 10 times at 65 °C (extrusion step, E).

### 2.4 Drug encapsulation analysis

Drug-loaded niosomes were separated from the free drug using centrifugation filters. Centrifugation filtration proved to be the superior method; it offered rapid processing without requiring dilution while successfully maintaining the structural integrity of the niosomes. We verified that the applied centrifugation force did not rupture the niosomes, as the concentration of the filtrate remained unchanged under various centrifugation force loadings and the niosomes remained in the similar size range prior to centrifugation. In addition to the employed centrifugation filtration, we tested other techniques to separate the free drug from the drug-loaded niosomes, *e.g.*, vacuum filtration, ultracentrifugation, gel filtration chromatography, and dialysis. We found a large variation between these methods compared to centrifugation filtration. These results are in agreement with the work of González-Menéndez *et al.*^38^ who also concluded that the chosen method plays a key role and may bias the obtained results. They identified centrifugation filtration as the most suitable approach, which is in agreement with our experience. To determine encapsulation efficiency, 0.5 mL of the niosomal suspension was loaded into Amicon Ultra 0.5 mL MWCO 3 kDa centrifugal filter units (Amicon, USA). Prior to use, the filters were pre-rinsed with deionized water and centrifugation was performed using an Eppendorf MiniSpin at 10 000 rpm (approximately 6700×*g*) for 20 min at a controlled room temperature (25 °C). DLS analysis confirmed that niosomes remained intact after centrifugation, with no significant changes observed in their mean diameter. The filtrate contained the free drug, which was detected using a Cary 60 UV-vis spectrophotometer (Agilent Technologies, USA). The concentration of a free drug was calculated based on the calibration curve, which describes the drug concentration as a function of absorbance at the peak absorbance wavelength *λ*_max_. For tetracaine and tetracaine HCl, the absorbance peaks were at 310 and 312 nm, respectively. The calibration curves can be found in the SI. This model drug was selected as there is no interference from the surfactant and cholesterol in this region of the spectrum. Finally, the encapsulation efficiency (EE) was calculated using the following formula:1
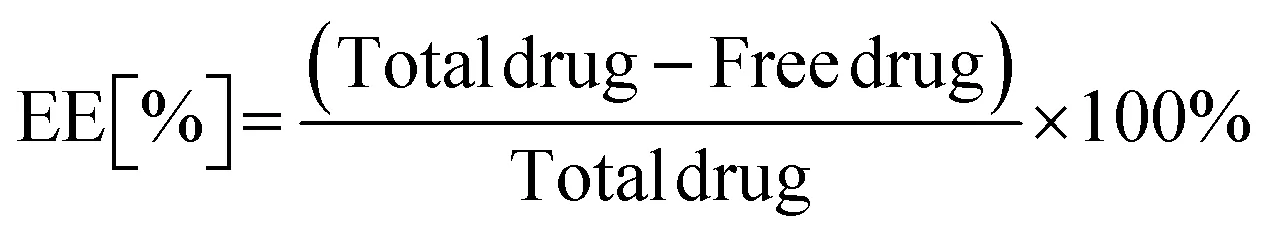


### 2.5 Characterization of niosomes

The hydrodynamic diameter and polydispersity index (PDI) of the niosomes were measured by dynamic light scattering (DLS) using an LS spectrometer with 2D pseudo-correlation mode (LS Instruments, Switzerland) at 25 °C. Prior to analysis, samples were diluted at 1 : 10 in deionized water to minimize multiple scattering effects. For the initial screening of niosomal preparation methods, scattered light was detected at three scattering angles (35°, 90°, and 140°) to evaluate the consistency of size measurements. The reported hydrodynamic diameter for these screening experiments corresponds to the average of the measurements obtained at the three angles. The variation among these measurements was used as an indicator of size distribution broadness. For all subsequent experiments, measurements were performed at a fixed scattering angle of 90°, and both the hydrodynamic diameter and PDI were recorded. Instrument laser intensity and detector settings were automatically set using the instrument software prior to each measurement. Scattering data were collected for 20 s and analyzed using the cumulant method.^39–41^ All measurements were performed in triplicate (*n* = 3), unless otherwise stated. Results are presented as mean ± standard deviation (SD).

Cryo-TEM analysis of the niosomes was used as a qualitative approach to confirm the morphology and vesicular structure of niosomes. The samples for cryo-TEM were prepared by plunge freezing. Briefly, a 4 µL sample was applied to a glow discharged Quantifoil cryoEM grid (R 2/1, 300 mesh) and incubated for 10 s in a Leica GP2 plunge freezer at room temperature and 80% relative humidity. The grid was then automatically blotted using filter paper for 4 s and frozen by immersion into liquid ethane cooled to −180 °C. The frozen grids were transferred under cryogenic conditions to the Simple Origin dual-grid cryo-holder (Model 215). Cryo-EM data were acquired in a semi-automated mode using SerialEM software^42^ on a Jeol JEM-2100Plus TEM microscope equipped with a LaB_6_ electron gun and TVIPS XF416 CMOS camera, operated at 200 kV. The data were collected at a pixel size of 2.31 Å per px, with an exposure time of 3.0 s and a total electron dose of 50 e Å^−2^ per micrograph. The nominal defocus was set to −3 µm. Denoising and contrast adjustment of the acquired images were performed using ImageJ software.^43^

### 2.6 Molecular dynamics simulations

The niosomal systems Span 60 and Tween 60 were prepared using the MemGen web server.^44^ We prepared four systems: neat Span 60 and Tween 60 + cholesterol in water and Span 60 and Tween 60 with 10 tetracaine molecules in neutral (TCN) and protonated (TCNH) form, respectively. The ratio between the surfactant and cholesterol was 3 : 1. The total number of Span 60 and Tween 60 surfactants was 150, with an additional 50 cholesterol molecules. Span 60 niosomal systems were hydrated with *ca.* 10 000 TIP3P water molecules, whereas Tween 60 systems were hydrated with *ca.* 25 000 TIP3P water molecules due to their larger size in *xy* dimensions than Span 60. Systems with protonated tetracaine (TCNH) were neutralized using chloride counterions. The obtained systems were first energy minimized, and then 500 ns long production runs for Span 60 and 1000 ns for Tween 60 systems were carried out in the presence of either 10 neutral tetracaine (TCN) or tetracaine chloride (TCNH) molecules, respectively. The first 100 ns were considered the equilibration period and disregarded from the analysis. The force field used for niosomes (Span 60 and Tween 60) was parameterized using the CGenFF force field,^45^ cholesterol and ions were described using the CHARMM36m force field,^46^ and water was modeled using the TIP3PS model.^47^ The models for Span 60, Tween 60, TCN and TCNH were built using Ligand Reader & Modeler module in CHARMM-GUI.^48,49^ For Tween 60, the length of poly(ethylenglycol) fragments (*x*, *y*, *w*, and *z*) is equal to 5. The equation of motion was solved using a 2 fs timestep and the leap-frog algorithm, with an updating frequency of 20 steps. The smooth particle mesh Ewald with a cut-off of 1.2 nm was used to treat electrostatic interactions.^50^ van der Waals interactions were treated using a cut-off of 1.2 nm, with the forces smoothly attenuated to zero between 1.0 and 1.2 nm. The temperature was kept constant at 298 K using the Nosé–Hoover thermostat with two coupling groups (niosome and the drug and remaining solvent with ions),^51,52^ while pressure was maintained at 1 bar with semi-isotropic coupling using the Parrinello–Rahman barostat.^53^ The time constants for coupling were set to 1 and 5 ps for the thermostat and barostat, respectively. All covalent bonds in niosomes and the drug molecules involving hydrogens were constrained using the P-LINCS algorithm.^54^ The SETTLE algorithm was used for water molecules.^55^ All simulations were performed using Gromacs 2022.^56^

## 3 Results and discussion

### 3.1 The size and vesicular structure of niosomes

Span and Tween surfactants are often reported as suitable compounds for the preparation of niosomal suspensions. Therefore, we used Span 60 and Tween 60 in combination with cholesterol as baseline formulations to verify the formation of niosomes at the selected precursor concentrations. Cryo-TEM imaging revealed rounded vesicular particles with a bilayer structure consistent with previously reported niosomal^57^ and liposomes^58^ systems. Cryo-TEM was used primarily as a qualitative method to confirm vesicle formation and morphology. For the tested Span 60 and Tween 60 surfactants, predominantly unilamellar niosomes were observed at a surfactant-to-cholesterol molar ratio of 3 : 1 when prepared using OPIm, as shown in Fig. 1. Span 60 niosomes exhibited mostly spherical and relatively uniform morphology, whereas Tween 60 formulations displayed not only spherical vesicles but also more irregularly rounded structures.

**Fig. 1:**
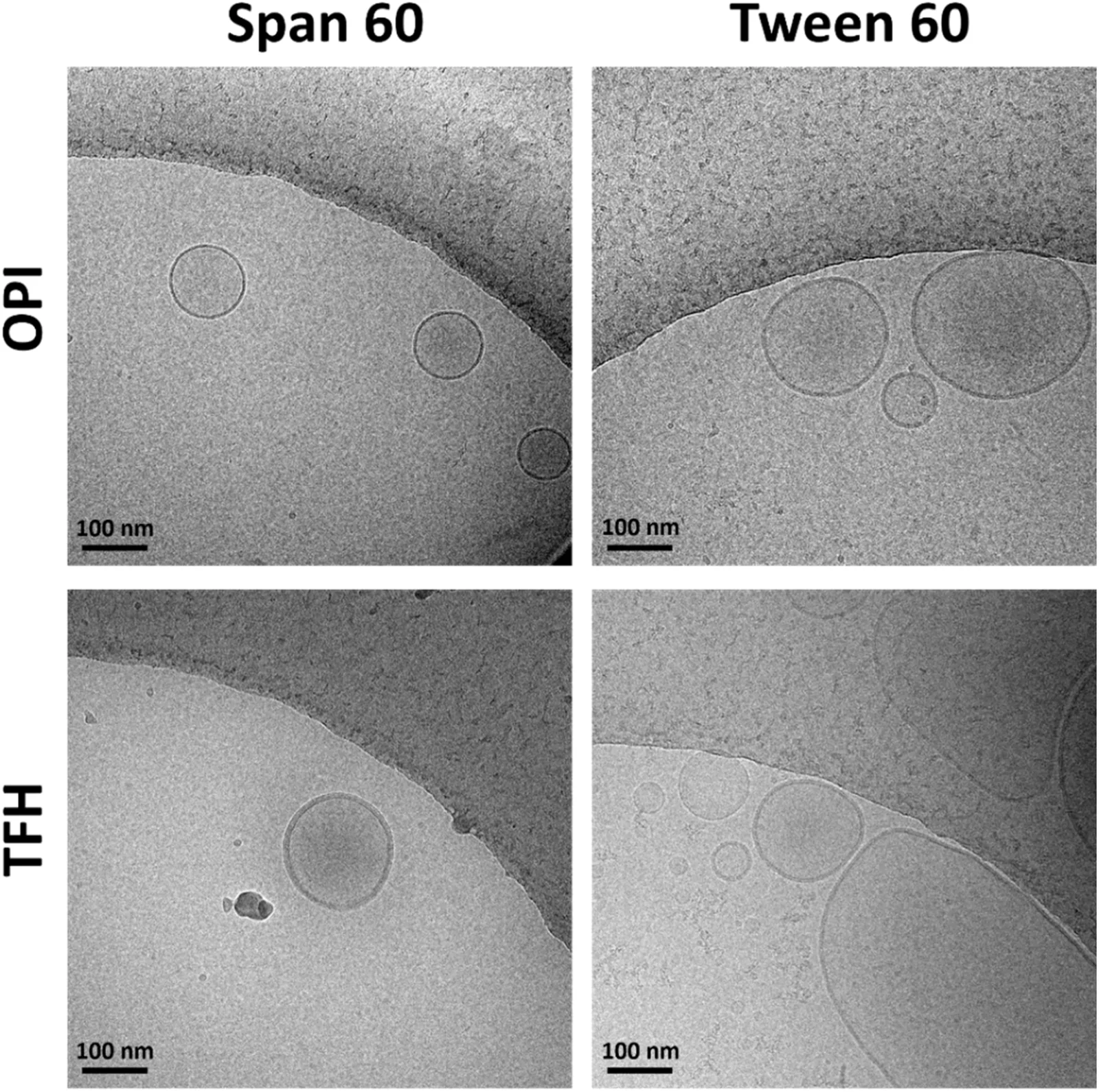
Cryo-TEM images of Span 60 and Tween 60-based niosomes with the addition of cholesterol at a molar ratio of 3 : 1 prepared using OPIm (top) and TFHm (bottom).

Based on the DLS results shown in Table 1, TFHm produced larger and more polydisperse niosomes compared to OPIm-prepared formulations. The influence of surfactant HLB on vesicle size was particularly apparent for TFHm, where the higher-HLB surfactant Tween 60 produced larger vesicles than Span 60. This trend was also qualitatively reflected in the cryo-TEM images in Fig. 1, where Tween 60 vesicles appeared less uniform and structurally more flexible compared to the more compact Span 60 vesicles. The observed differences are likely related to the higher hydration and increased bilayer fluidity associated with Tween 60, whereas the lower-HLB Span 60 forms a more tightly packed bilayer structure.

**Table 1:** Preparation of plain Span 60 and Tween 60 niosomes, showing the organic phase used, reaction temperature, HLB values, and average vesicle size measured by dynamic light scattering (DLS) at a scattering angle of 90°

Surfactant	Organic phase	Aqueous phase temperature for OPI [°C]	HLB	Average size (nm)	
				Organic phase injection (OPI)	Thin film hydration (TFH)
Span 60	Isopropyl alcohol	90	4.7	201 ± 67	646 ± 462
Tween 60	Diethyl ether	55	14.9	251 ± 199	1108 ± 229

The vesicle dimensions observed by cryo-TEM were in qualitative agreement with the DLS measurements. However, some differences between the apparent sizes obtained by the two techniques are expected due to their different measurement principles. DLS measures the hydrodynamic diameter of particles dispersed in a solution, including the surrounding hydration layer, whereas cryo-TEM visualizes vitrified vesicles in the frozen state. Therefore, DLS was considered the primary quantitative method for vesicle size determination, while cryo-TEM mainly served to confirm vesicular morphology and bilayer organization.

### 3.2 Effect of freeze–thaw cycles and extrusion on encapsulation efficiency

Water-soluble tetracaine HCl (TCNH) and organic phase-soluble tetracaine (TCN) were selected as model drugs with similar molecular weight but different affinity to the aqueous phase. They were incorporated into niosomes using TFHm and OPIm. An extrusion step can be employed after the preparation of niosomes to decrease their size and lamellarity with the aim of increasing the encapsulation potential and decreasing the polydispersity.^35^ Furthermore, the freezing step, which leads to niosomal rupture, and the thawing step recreating the niosomes were reported to positively influence the EE.^35,59^ Therefore, we used Span 60-based niosomes to assess this method and applied E after the initial preparation as well as FT + E to observe its influence on the drug EE. We tested 2, 5, and 10 FT cycles followed by the E step. The FT step is responsible for rupturing and reassembling the niosomal bilayer, which is strongly related to the niosomal size; hence, we monitored both the evolution of the EE and the hydrodynamic diameter of the niosomes.

The highest EE of TCNH was 16.1 ± 7.7% using OPIm and 17.8 ± 3.0% using TFHm (Fig. 2). For both preparation methods, the highest average EE of tetracaine HCl was obtained after 2× FT cycles followed by extrusion (E). The FT procedure alone did not increase the EE of TCNH unless followed by extrusion. Increasing the number of FT cycles to 5× FT and 10× FT did not result in a further increase in TCNH encapsulation efficiency. Overall, the EE of water-soluble tetracaine HCl was substantially lower than that of organic phase-soluble tetracaine for both TFHm and OPIm formulations.

**Fig. 2:**
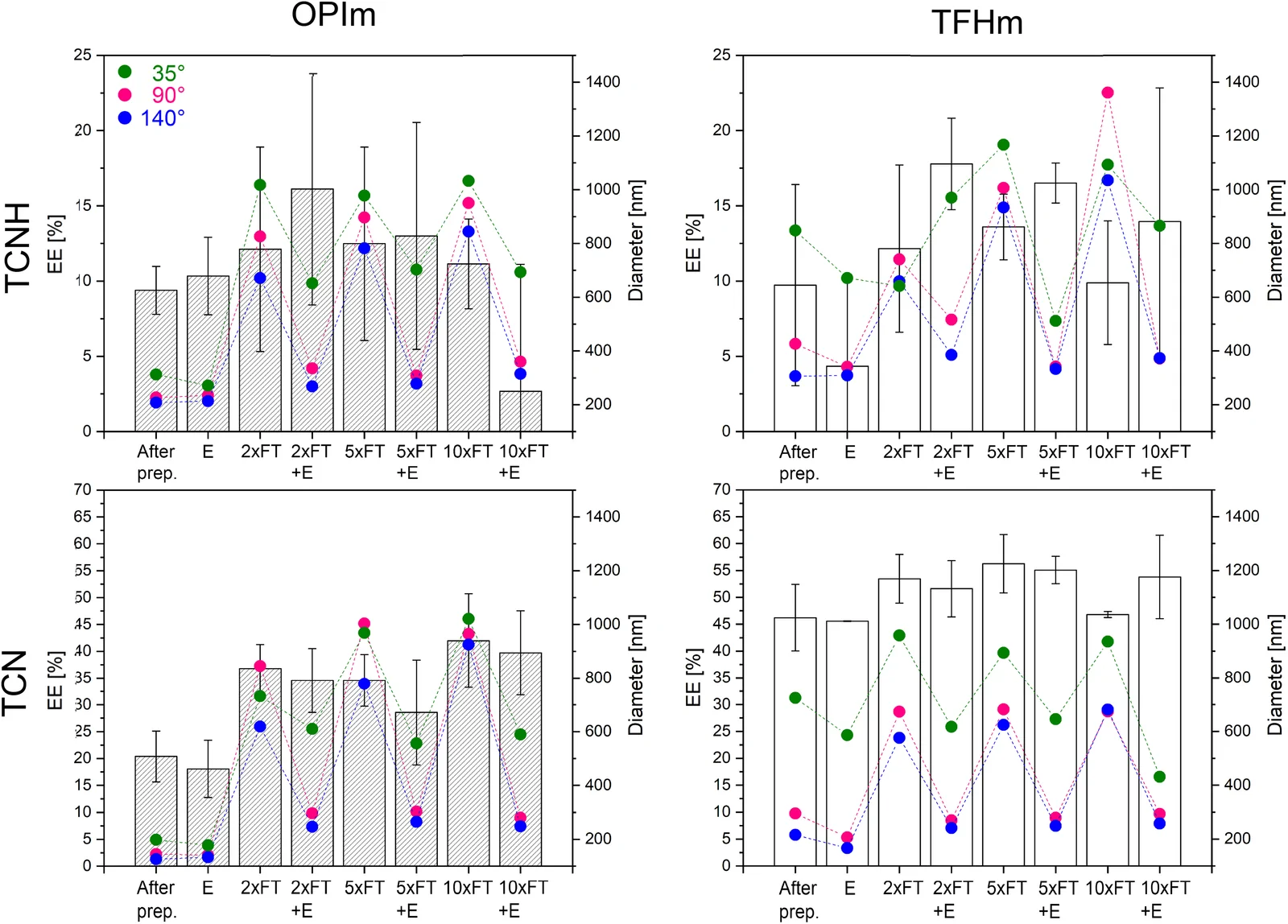
Both tetracaine HCl (TCNH) and tetracaine (TCN) were encapsulated in Span 60 niosomes using THFm and OPIm. Encapsulation efficiency was measured after the initial preparation, the E step, and after 2, 5 and 10 FT cycles each accompanied by the E step. Niosomal sizes were measured using DLS at 35°, 90°, and 140° angles to detect the largest, medium, and the smallest vesicles, respectively. The experiments were performed three times.

In the case of TCN, 2× FT cycles increased the EE from 20.4% to 36.8% when OPIm was used. In contrast, only a minor effect of FT + E treatment on TCN encapsulation was observed for TFHm-prepared niosomes. The highest EE values of TCN were 36.8 ± 4.5% for OPIm after 2× FT cycles and 56.2 ± 5.4% for TFHm after 5× FT cycles (Fig. 2). The extrusion step applied after FT slightly decreased the EE of tetracaine, whereas a slight increase was observed for TCNH.

### 3.3 Effect of freeze–thaw cycles and extrusion on niosome size

In parallel with the EE measurements, the evolution of niosomal size was measured at scattering angles of 35°, 90°, and 140° to evaluate the effect of the FT + E process on vesicle size distribution (Fig. 2). The FT cycles applied after niosome preparation increased the vesicle diameter in all tested formulations due to disruption and subsequent reformation of the niosomal bilayer. In contrast, the subsequent extrusion step reduced the vesicle size as the dispersions passed through the defined porous membrane of the lipid extruder. This cyclic increase and decrease in vesicle size was consistently observed following the alternating FT and extrusion procedures.

Although the size of niosomes after FT + E was close to the initial value measured after their preparation, the fraction of the largest niosomes (measured at an angle of 35°) remained present and was not suppressed by E. This size evolution, comprising the size increases after FT and decreases after E, was observed for both TCNH and TCN and for both preparation methods including OPIm and TFHm. Although the trend was similar, there was an increase in the number of large niosomes present immediately after the preparation using TFHm, unlike OPIm, which is represented by the increased diameter of the niosomes measured at an angle of 35°. The E step applied after the niosomal preparation using TFHm did not fully unify the size distribution. In contrast, the size of the largest niosomes prepared using OPIm was increased as a result of the FT procedure, which equalized both methods regarding the largest fraction of the present niosomes.

In general, the EE values reported in the literature fluctuate between very low values up to the complete encapsulation of the added model compound. The preparation method, encapsulated compound, surfactant used, addition of the supporting agent, or its amount, all have an impact on the EE. Furthermore, Gonzalez Gomez *et al.*^60^ also pointed out the importance of the separation technique that can influence the EE results.

### 3.4 Influence of aqueous phase pH on encapsulation efficiency

To the best of our knowledge, no data suitable for comparison with our system were available in the literature regarding the encapsulation of tetracaine or tetracaine HCl in niosomes. However, tetracaine HCl and tetracaine used in this work have a molecular structure close to that of lidocaine HCl and lidocaine, which were encapsulated in Tween 20-based niosomes by Carafa *et al.*^27^ They reached a similar EE for both the free base and HCl form at a pH of 5.5. In addition, Carafa *et al.*^27^ focused on the influence of pH during the encapsulation of lidocaine and lidocaine HCl in Tween 20-based niosomes. They observed comparable EE values for both the free base and HCl when the aqueous buffered phase with a pH of 5.5 was used. For the increase in pH to 8.6, the encapsulated concentration of both forms was negligible. Therefore, we decided to further investigate the influence of pH of the aqueous phase on the EE of tetracaine HCl and tetracaine (see Table 2). Tetracaine is a weak base with a reported p*K*_a_ of approximately 8.5, indicating that the protonated form predominates under acidic conditions, whereas the neutral free base becomes increasingly dominant at alkaline pH values.^61^ Since the ionization state strongly influences the affinity of tetracaine toward the hydrophobic niosomal bilayer, changes in pH are expected to affect its encapsulation behavior. We chose OPIm for subsequent experiments as the vesicle sizes for Span 60 and Tween 60 were similar (approximately 200–300 nm). This consistency in size ensures comparable experimental conditions, making it more suitable for molecular dynamics simulation study.

**Table 2:** The EE values of TCN and TCNH in OPIm prepared niosomes at different pH values of the aqueous phase. All experiments and analysis were conducted three times

pH (aqueous phase)	Span 60		Tween 60	
	TCN	TCNH	TCN	TCNH
	EE [%]	EE [%]	EE [%]	EE [%]
5.3	39.8 ± 3.2	2.8 ± 0.5	54.9 ± 2.9	6.5 ± 3.1
6.3	26.7 ± 3.0	9.4 ± 1.6	68.9 ± 4.8	3.8 ± 2.4
7.4	20.4 ± 4.7	19.7 ± 1.6	65.1 ± 3.2	0.2 ± 0.0
9.3	34.6 ± 3.1	30.5 ± 2.1	56.5 ± 3.8	44.6 ± 3.4
11	33.4 ± 2.8	41.3 ± 0.7	67.5 ± 4.1	74.0 ± 3.1

We observed that the EE of TCNH in both surfactant systems increased with increasing pH. In contrast, no clear trend in EE was observed for TCN across the investigated pH range. Tween 60 formulations generally exhibited higher EE values for TCN compared with Span 60 formulations, which may be associated with the higher HLB and increased bilayer fluidity of Tween-based vesicles. The more flexible bilayer structure may facilitate partitioning of tetracaine within the membrane region during vesicle formation. The enhanced encapsulation of TCN compared to TCNH can be explained by the interaction with the niosomal bilayer during the preparation. Tetracaine, being present in the organic phase of the preparation, has less contact with the aqueous environment during the vesiculation process. Furthermore, tetracaine is supposed to be primarily encapsulated within the niosomal bilayer, which could protect the compound from the external aqueous environment. In contrast, TCNH was dissolved in the aqueous phase during the preparation step. That leads to a direct impact of pH on the drug transformation from HCl to the free base form during its accommodation in the niosomal aqueous core. As the pH increased from 5.3 to 11, progressive deprotonation of TCNH resulted in the formation of the neutral tetracaine base, which exhibits greater affinity toward the hydrophobic bilayer environment. This transition was accompanied by increased encapsulation efficiency for both surfactant systems. These findings highlight the influence of pH, drug properties, and HLB of surfactant on improving the encapsulation efficiency of the niosomes.

### 3.5 Colloidal stability of suspensions

In addition to encapsulation efficiency, vesicle size, polydispersity index (PDI), and zeta potential were also assessed to evaluate the structural characteristics and colloidal stability of the prepared niosomes. For Span 60 niosomes loaded with TCN (as seen in Table 3), the vesicle sizes remained within the 130–170 nm range across all pH values. In contrast, TCNH-loaded Span 60 niosomes ranged from 130–230 nm, with larger vesicles present at lower pH. At higher pH, TCNH undergoes deprotonation and converts to neutral TCN, which has greater affinity for the niosomal membrane. As a result, vesicle sizes decrease and become more comparable to those of the TCN-loaded formulation.

**Table 3:** The hydrodynamic diameters (measured by DLS at a scattering angle of 90°) and PDI of TCN and TCNH in Span 60 OPIm prepared niosomes at different pH values of the aqueous phase. All measurements were performed in triplicate (*n* = 3) and results are expressed as mean ± standard deviation

pH	Span 60			
	TCN		TCNH	
	Diameter (nm)	PDI	Diameter (nm)	PDI
5.3	159.3 ± 4.3	0.30 ± 0.16	230.0 ± 20.2	0.20 ± 0.17
6.3	151.6 ± 8.9	0.22 ± 0.01	220.9 ± 5.4	0.14 ± 0.13
7.4	129.2 ± 2.4	0.18 ± 0.03	176.3 ± 18.2	0.36 ± 0.23
9.3	129.6 ± 4.7	0.19 ± 0.11	128.6 ± 5.7	0.38 ± 0.06
11	167.0 ± 9.4	0.21 ± 0.13	134.8 ± 11.2	0.20 ± 0.09

The hydrodynamic diameters of Tween 60 niosomes (Table 4) exhibited greater variability across pH values compared to Span 60 formulations, reflecting the higher HLB and increased membrane fluidity of Tween-based vesicles. TCN-loaded niosomes showed sizes ranging from approximately 127 to 194 nm. TCNH-loaded vesicles displayed a similar trend, with diameters between 143 and 192 nm. Several Tween 60 formulations exhibited PDI values approaching 0.5–0.6, indicating relatively broad vesicle size distributions and increased heterogeneity within the dispersions. This behavior is consistent with the less rigid and more fluid bilayer structure associated with high-HLB surfactants and suggests that Tween 60 systems may exhibit lower structural uniformity compared with Span 60 formulations.

**Table 4:** The hydrodynamic diameters (measured by DLS at a scattering angle of 90°) and PDI of TCN and TCNH in Tween 60 OPIm prepared niosomes at different pH values of the aqueous phase. Results are expressed as mean ± standard deviation (*n* = 3)

pH	Tween 60			
	TCN		TCNH	
	Diameter (nm)	PDI	Diameter (nm)	PDI
5.3	127.0 ± 8.2	0.26 ± 0.12	143.2 ± 5.9	0.49 ± 0.07
6.3	194.2 ± 12.2	0.53 ± 0.07	164.5 ± 12.7	0.43 ± 0.05
7.4	151.5 ± 11.5	0.59 ± 0.10	148.7 ± 8.7	0.51 ± 0.01
9.3	160.8 ± 6.3	0.50 ± 0.07	191.7 ± 5.3	0.39 ± 0.03
11	170.4 ± 18.5	0.42 ± 0.02	153.0 ± 4.3	0.41 ± 0.03

The zeta potentials of both Span 60 and Tween 60 niosomes were strongly negative across the investigated pH range (Table 5), suggesting electrostatic contributions to dispersion stability. However, colloidal stability is also influenced by vesicle size distribution, bilayer composition, and polydispersity, particularly in formulations exhibiting elevated PDI values. Moreover, Span 60 niosomes loaded with TCN showed an extremely negative zeta potential of −78 mV at pH 11, whereas an increase in the negative zeta potential with increasing pH was evident for Tween 60 niosomes loaded with TCNH, with lower zeta potentials measured at pH 5.3 and 6.3 (−18.6 mV and −16.0 mV). The zeta potential trends reflect the pH-dependent ionization of tetracaine, with the protonated form reducing surface negativity at low pH and neutral form enhancing negativity at higher pH.

**Table 5:** Zeta potentials of Span 60 and Tween 60 formulations with TCN and TCNH in different pH environments. Results are presented as (*n* = 3) mean ± standard deviation

pH (aqueous phase)	Span 60		Tween 60	
	TCN	TCNH	TCN	TCNH
	mV	mV	mV	mV
5.3	−45.7 ± 0.4	−51.9 ± 1.4	−39.9 ± 0.6	−18.6 ± 0.5
6.3	−51.4 ± 4.3	−47.7 ± 1.6	−43.7 ± 1.6	−16.0 ± 0.9
7.4	−39.4 ± 1.3	−55.9 ± 3.4	−34.1 ± 1.4	−28.3 ± 2.0
9.3	−44.7 ± 3.0	−58.9 ± 4.9	−38.4 ± 0.3	−32.3 ± 0.6
11	−77.8 ± 1.8	−48.3 ± 3.2	−36.7 ± 1.4	−41.3 ± 1.0

### 3.6 Molecular dynamics simulations

In order to understand the observed phenomena on an atomistic level, we performed detailed MD simulations of the bilayer for both Span 60 and Tween 60 surfactants in water or in the presence of tetracaine (TCN) or tetracaine HCl (TCNH). The chemical structures of the surfactants and API can be found in Fig. 3.

**Fig. 3:**
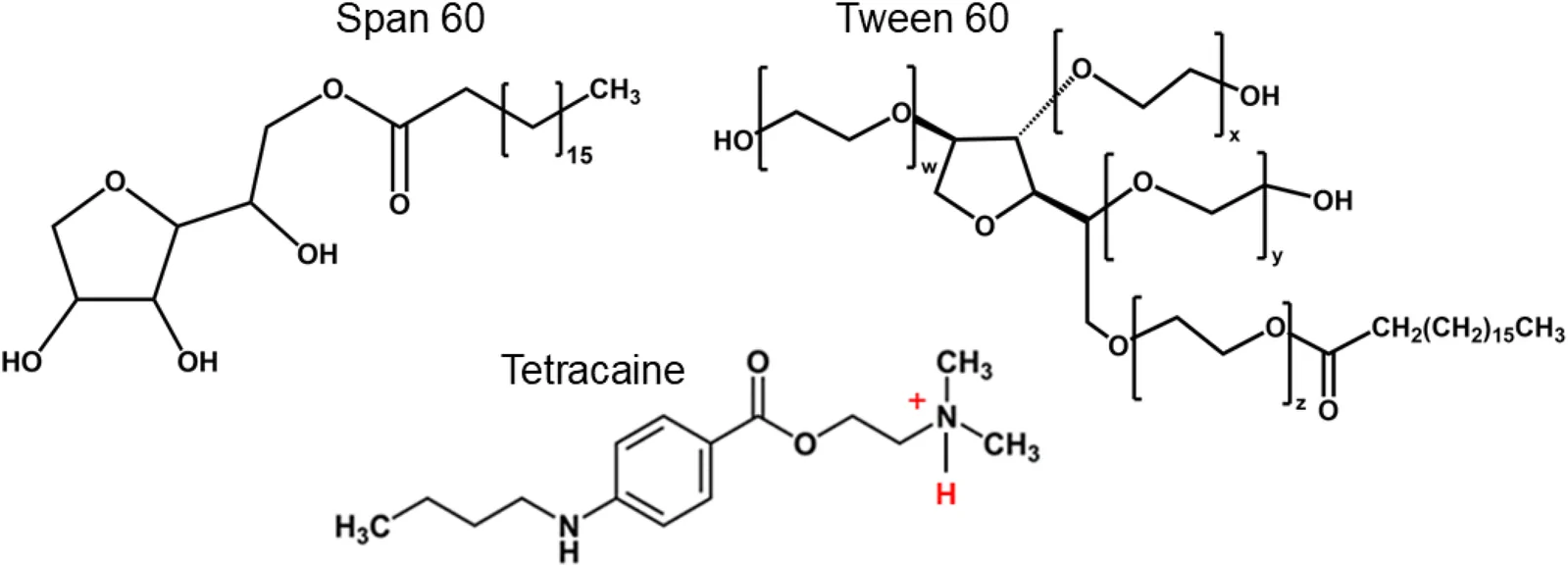
Span 60, Tween 60 (indices *x*, *y*, *w*, and *z* are equal to 5), and tetracaine (and its protonated form).

The results obtained for surfactant bilayers in water in terms of the number density profiles of Span 60 or Tween 60 are presented in Fig. 4.

**Fig. 4:**
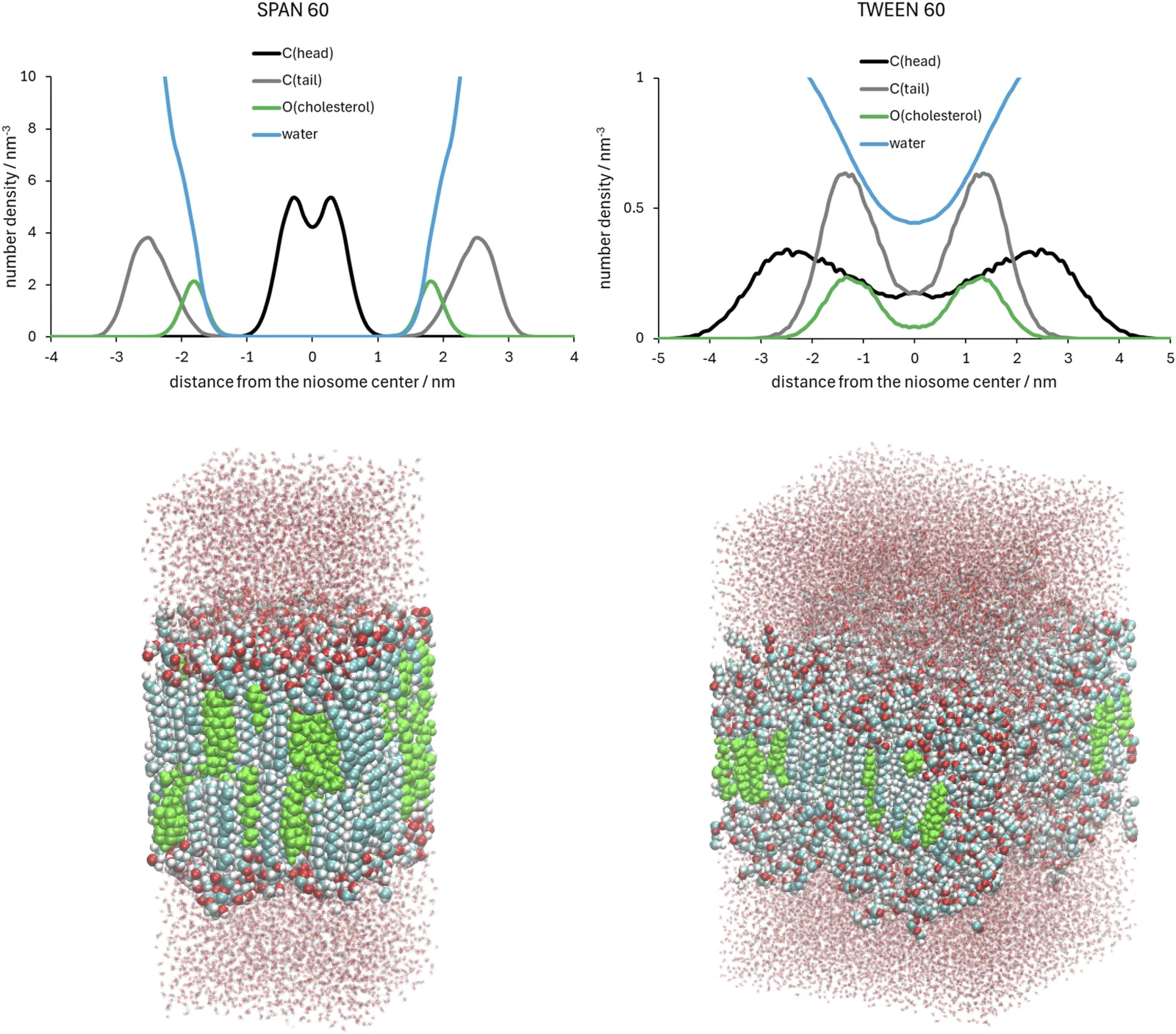
Top panel: number density profiles of Span 60 and Tween 60 systems (top). Surfactant head carbon atoms are shown in black, and tail carbon atoms are shown in grey. Cholesterol is shown in green, and water is shown in blue. The number density of water is reduced by 50× for Tween 60. Bottom panel: two selected snapshots from MD simulations for Span 60 and Tween 60 (bottom). Surfactant molecules (Span 60 or Tween 60) are shown in vdW representation. Cholesterol is shown in green vdW representation, and water is shown as transparent lines in the vicinity of the niosome.

From the data presented in Fig. 4, we see that the structures of the niosomal bilayers are markedly different for each surfactant. Specifically, due to its low HLB value of 4.7, the Span 60 niosomal bilayer is compact, and the interior is essentially not hydrated, as evidenced by negligible water density in the niosomal center. Its area per surfactant molecule is 0.249 nm^2^, which is significantly smaller than that of a typical lipid bilayer, such as POPC (0.643 nm^2^, ref. 62). This is not surprising since the surfactant hydrophobic tails of Span 60 possess a single hydrophobic chain, thus enabling tight packing in the hydrophobic interior. The results generally agree with previous MD studies for mixed Span 60/cholesterol systems.^63^

On the other hand, the structure of the Tween 60 bilayer, which has an HLB value of 14.9, is significantly different from that of the Span 60. Specifically, the area per surfactant molecule is much larger, being 0.832 nm^2^, which is significantly larger than that of the typical POPC bilayer. This is the consequence of its larger hydration, not only of the surfactant headgroup but also of the whole niosome, demonstrated by the high number density of water, which in turn leaks across the niosomal bilayer freely, in strong contrast to weakly hydrated Span 60. Consequently, the individual surfactant molecules or their combination with cholesterol can also flip-flop across the bilayer, creating a highly dynamic complex structure. Still, despite the strong hydration and free movement of the surfactant molecules, the structure of the Tween 60 bilayer is remarkably stable at the hundreds of nanosecond timescale in our MD simulations. This unusual hydration of Tween 60 is in accordance with the high HLB value (Table 1). All-atom MD simulations for the Tween 60 : cholesterol (3 : 1) system have not been published so far, to the best of our knowledge. However, the results for neat Tween 60 systems, without cholesterol, were not stable (as in our experiments) while a stable bilayer was formed in the system with the 1 : 1 ratio (Tween 60 : cholesterol).^64^

Fig. 5 and 6 show the number density profiles of Span 60 and Tween 60 niosomes upon the addition of finite concentration of either neutral tetracaine (TCN) during thin lipid formation or tetracaine chloride (TCNH) *via* aqueous solution.

**Fig. 5:**
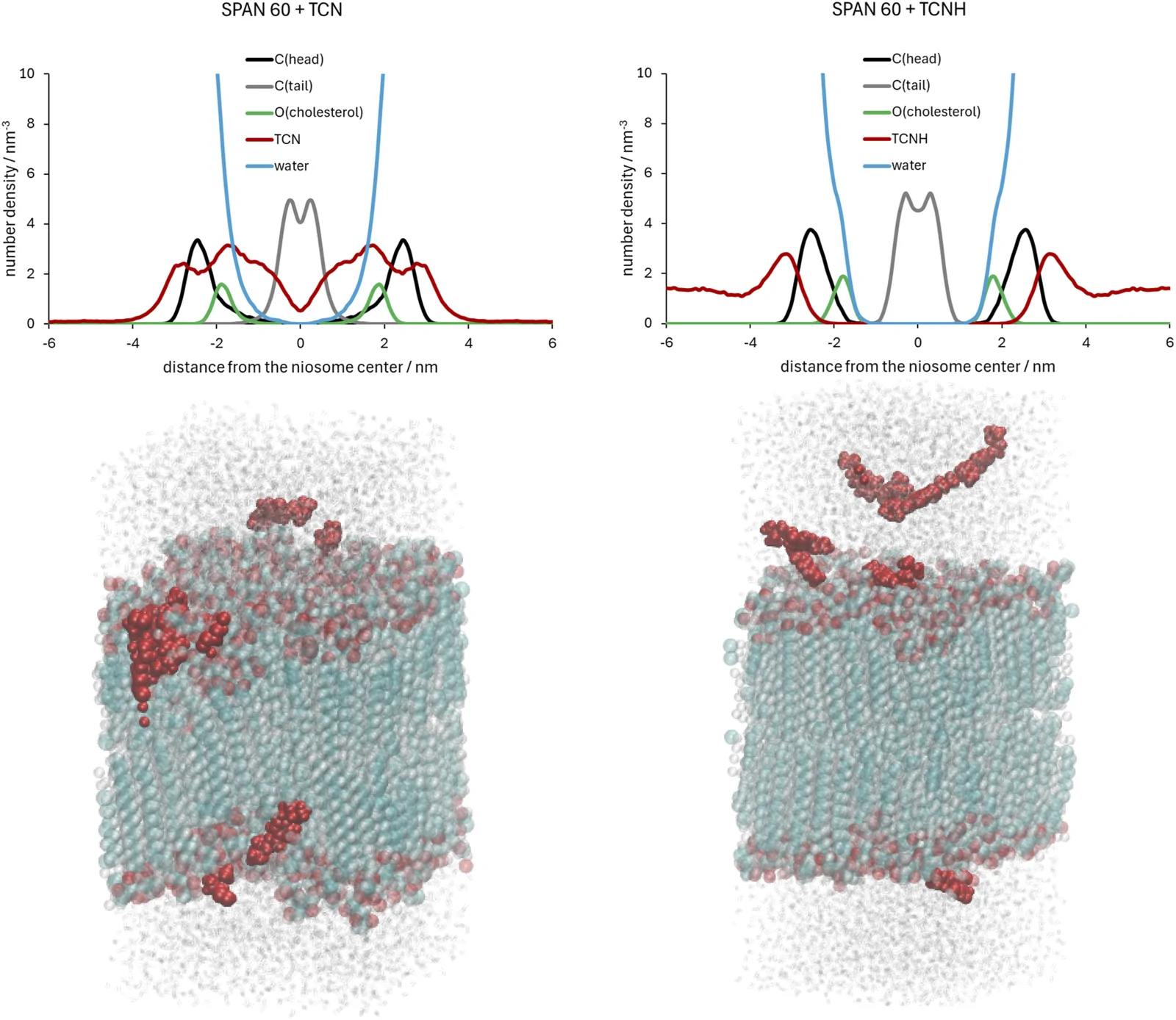
Top panel: number density profiles of Span 60 niosomes with the addition of a finite concentration of either neutral (TCN) or protonated tetracaine (TCNH) drug. Surfactant head carbon atoms are shown in black, and tail carbon atoms are shown in grey. Cholesterol is shown in green, TCN molecules are shown in dark red, and water is shown in blue. Bottom panel: two selected snapshots from MD simulations for Span 60 and the TCN/TCNH drug. Surfactant molecules and cholesterol are shown in transparent vdW representation. The drug (TCN or TCNH) is shown in dark red vdW representation, and water is shown as ghost lines in the vicinity of the niosome.

**Fig. 6:**
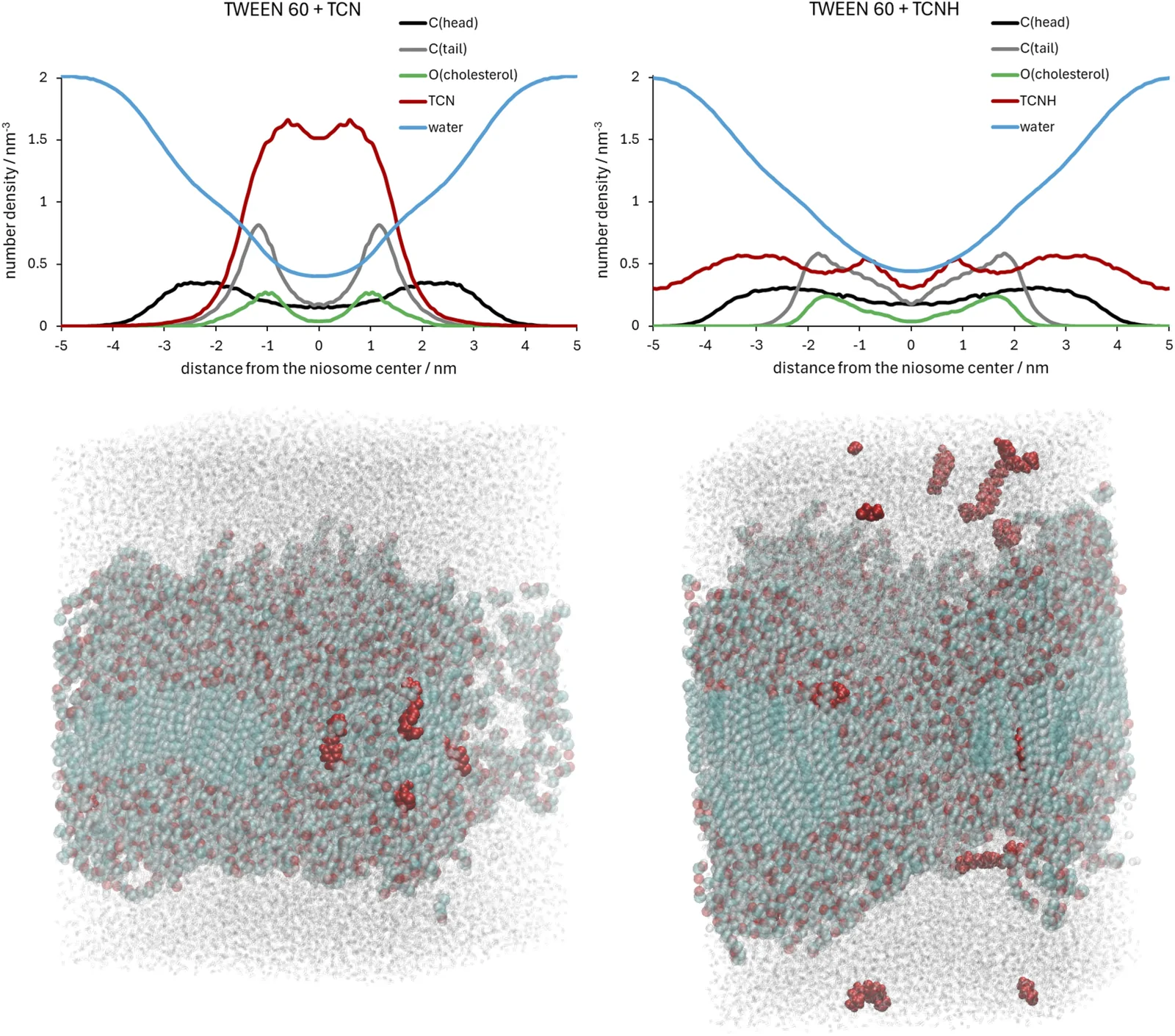
Top panel: number density profiles of Tween 60 niosomes with the addition of a finite concentration of either neutral (TCN) or protonated tetracaine (TCNH) drug. Surfactant head carbon atoms are shown in black, and tail carbon atoms are shown in grey. Cholesterol is shown in green, TCN molecules are shown in dark red, and water is shown in blue. Bottom panel: two selected snapshots from MD simulations for Tween 60 and the TCN/TCNH drug. Surfactant molecules and cholesterol are shown in transparent vdW representation. The drug (TCN or TCNH) is shown in dark red vdW representation, and water is shown as ghost lines in the vicinity of the niosome.

When focusing on the number concentration profile of the neutral TCN, it is visible that, for both niosomes, the drug is efficiently captured inside the niosome. This qualitatively agrees with the high encapsulation efficiency (EE) of Tween 60, which is more than 60%, whereas in the case of Span 60 it is around 20% at neutral pH (Table 2).

However, when TCN is added from the aqueous phase as a chloride salt, the EE values for Tween 60 are almost negligible, whereas the EE for Span 60 remains similar to the EE of the neutral drug (around 20% at pH 7.4). In the case of Span 60, the drug is not located inside the niosome, but instead at the niosome/water interface in the polar region of the Span 60 surfactant, as evidenced by the maximum average distance of around 1.5 nm from the niosome center. On the other hand, the negligible EE for TCNH in the Tween 60 niosomes is a consequence of two factors: first, it cannot efficiently stabilize inside the niosome (or at the niosome/water interface), which is evidenced by a significant amount of TCNH in the aqueous phase. Furthermore, TCNH undergoes a facile diffusion across the niosome, as evidenced by the comparable number density in the niosome *vs.* water phase. This is the consequence of the high hydration of the Tween 60 niosomal bilayer and the existence of transient water pores where the TCNH cation, a relatively small molecule, diffuses together with water across the bilayer. In the case of Span 60 niosomes, TCNH cannot diffuse along the bilayer (the number density of the drug is zero in the niosomal center) due to a less hydrated interior, thus explaining the increased EE efficiency compared to Tween 60 niosomes.

Finally, we can connect the pH-dependent values of EE and different niosome preparation methods with the MD simulation results. In the cases where the neutral tetracaine is added directly to the nonpolar film, *i.e.*, after the TFHm procedure, the EE is essentially not dependent on the pH value. This is expected, since during TFHm, the drug remains neutral in the niosome bilayer without contact with the aqueous phase added on top of the film. Therefore, the encapsulation efficiency values for the TFHm are relatively high for both Tween 60 (>50%) and Span 60 systems (>20%) at all pH values of the surrounding aqueous solution. On the other hand, when the tetracaine HCl drug is added *via* aqueous solution (the OPIm procedure), it is highly dependent on the pH value of the surrounding medium. Specifically, at lower pH values, where the drug is dominantly protonated, it will not efficiently embed in the membrane, as shown in Fig. 5 and 6. At higher pH values, especially above the p*K*_a_ value of tetracaine, which is 8.5, the dominant form of the drug is the neutral form, which easily inserts into the membrane, thus increasing the encapsulation efficiency, in agreement with MD simulation data. In the case of Span 60 systems, we see a linear increase in EE with pH (Table 2) for tetracaine chloride (TCNH) added *via* aqueous solution *via* the OPIm procedure. However, in the case of Tween60, the addition of TCNH at lower pH (where the tetracaine is protonated) results in negligible EE values since it will cross the niosome easily as described above. However, tetracaine will deprotonate at higher pH, and the measured EE is similar to that obtained by the TFHm procedure, where the drug is neutral from the start of the procedure. It is important to note that using all-atom molecular dynamics simulations for the analysis of surfactant systems with polymer-like PEG chains, such as Tween 60, is still quite limited due to deficiencies in the force-fields, which were not general for polymers. In this work, we used the CGenFF force field, which showed an excellent performance for general molecules,^45^ and we believe that it reliably captures the qualitative behavior of complex surfactants like Tween 60.

## 4 Conclusion

Niosomes formed from Span 60 and Tween 60 were prepared using TFHm and OPIm in this work. The vesicular shape of the niosomes was analyzed using cryo-TEM, and the size distribution was measured by angle-dependent DLS. Specifically, OPIm produced smaller niosomes compared to TFHm. TCN and TCNH were encapsulated in niosomal formulations composed of either Span 60 or Tween 60. Our results demonstrate that the encapsulation efficiency of TCNH in niosomes was significantly influenced by pH, while TCN encapsulation was affected by the surfactant type. Tween 60 niosomes provided better encapsulation in general at higher pH compared to Span 60 niosomes. The MD simulations revealed significant differences in the Tween 60 and Span 60 bilayer structure. Tween 60 consisted of a hydrated and dynamic bilayer, facilitating the diffusion of the tetracaine molecule across the bilayer, whereas the Span 60 bilayer was more rigid and non-hydrated. These findings have a potential application in niosomal drug delivery development as the encapsulation of ionizable drugs could be improved by tuning their pH and selecting an appropriate surfactant.

## Conflicts of interest

There are no conflicts to declare.

## Supplementary Material

NA-008-D6NA00065G-s001

## Data Availability

The data will be available on the Zenodo repository under https://doi.org/10.5281/zenodo.18170802, once the publication is accepted for publication. Supplementary information (SI) is available. See DOI: https://doi.org/10.1039/d6na00065g.

## References

[cit1] Ag Seleci D., Seleci M., Walter J.-G., Stahl F., Scheper T. (2016). Niosomes as Nanoparticular Drug Carriers: Fundamentals and Recent Applications. J. Nanomater..

[cit2] Luo Z.-H., He X.-Y., Deng N.-N. (2025). Monodisperse Giant Unilamellar Niosomes as Minimal Synthetic Cells. J. Am. Chem. Soc..

[cit3] Bhardwaj P., Tripathi P., Gupta R., Pandey S. (2020). Niosomes: A review on niosomal research in the last decade. J. Drug Delivery Sci. Technol..

[cit4] Marianecci C., Di Marzio L., Rinaldi F., Celia C., Paolino D., Alhaique F., Esposito S., Carafa M. (2014). Niosomes from 80s to present: The state of the art. Adv. Colloid Interface Sci..

[cit5] Handjani-Vila R. M., Ribier A., Rondot B., Vanlerberghie G. (1979). Dispersions of lamellar phases of non-ionic lipids in cosmetic products. Int. J. Cosmet. Sci..

[cit6] Handjani R. M. , Ribier A., Vanlerberghe G., Zabotto A. and Griat J., Cosmetic and pharmaceutical compositions containing niosomes and a water-soluble polyamide, and a process for preparing these compositions, US4830857A, 1989. https://patents.google.com/patent/US4830857A/en

[cit7] The Triumphs Of Formulation, L'Oréal, accessed online (23 Nov. 2021), https://www.loreal.com/en/articles/science%E2%88%92and%E2%88%92technology/the%E2%88%92triumphs%E2%88%92of%E2%88%92formulation/

[cit8] Allen T. M., Cullis P. R. (2013). Liposomal drug delivery systems: from concept to clinical applications. Adv. Drug Delivery Rev..

[cit9] Baillie A. J., Florence A. T., Hume L. R., Muirhead G. T., Rogerson A. (1985). The preparation and properties of niosomes – non-ionic surfactant vesicles. J. Pharm. Pharmacol..

[cit10] Yasamineh S., Yasamineh P., Ghafouri Kalajahi H., Gholizadeh O., Yekanipour Z., Afkhami H., Eslami M., Hossein Kheirkhah A., Taghizadeh M., Yazdani Y., Dadashpour M. (2022). A state-of-the-art review on the recent advances of niosomes as a targeted drug delivery system. Int. J. Pharm..

[cit11] Alyami H., Abdelaziz K., Dahmash E. Z., Iyire A. (2020). Nonionic surfactant vesicles (niosomes) for ocular drug delivery: Development, evaluation and toxicological profiling. J. Drug Delivery Sci. Technol..

[cit12] Antonara L., Triantafyllopoulou E., Chountoulesi M., Pippa N., Lagopati N., Dallas P. P., Rekkas D. M., Gazouli M. (2025). Recent advances in niosome-based transdermal drug delivery systems. Curr. Opin. Biomed. Eng..

[cit13] Purohit S. J., Tharmavaram M., Rawtani D., Prajapati P., Pandya H., Dey A. (2022). Niosomes as cutting edge nanocarrier for controlled and targeted delivery of essential oils and biomolecules. J. Drug Delivery Sci. Technol..

[cit14] Mahale N. B., Thakkar P. D., Mali R. G., Walunj D. R., Chaudhari S. R. (2012). Niosomes: Novel sustained release nonionic stable vesicular systems — An overview. Adv. Colloid Interface Sci..

[cit15] Bayindir Z. S., Yuksel N. (2010). Characterization of niosomes prepared with various nonionic surfactants for paclitaxel oral delivery. J. Pharm. Sci..

[cit16] Jadon P. S., Gajbhiye V., Jadon R. S., Gajbhiye K. R., Ganesh N. (2009). Enhanced Oral Bioavailability of Griseofulvin *via* Niosomes. AAPS PharmSciTech.

[cit17] Kushnazarova R., Mirgorodskaya A., Bushmeleva K., Vyshtakalyuk A., Lenina O., Petrov K., Zakharova L. (2024). Improving the Stability, Water Solubility, and Antioxidant Activity of α‑Tocopherol by Encapsulating It into Niosomes Modified with Cationic Carbamate-Containing Surfactants. Langmuir.

[cit18] Tadros T. F. , Introduction, in Applied Surfactants: Principles and Applications, Wiley-VCH, Weinheim, 2005, pp. 1–17

[cit19] Uchegbu I. F., Florence A. T. (1995). Non-ionic surfactant vesicles (niosomes): Physical and pharmaceutical chemistry. Adv. Colloid Interface Sci..

[cit20] Junyaprasert V. B., Singhsa P., Suksiriworapong J., Chantasart D. (2012). Physicochemical properties and skin permeation of Span 60/Tween 60 niosomes of ellagic acid. Int. J. Pharm..

[cit21] Yoshioka T., Sternberg B., Florence A. T. (1994). Preparation and properties of vesicles (niosomes) of sorbitan monoesters (Span 20, 40, 60 and 80) and a sorbitan triester (Span 85). Int. J. Pharm..

[cit22] Manosroi A., Wongtrakul P., Manosroi J., Sakai H., Sugawara F., Yuasa M., Abe M. (2003). Characterization of vesicles prepared with various non-ionic surfactants mixed with cholesterol. Colloids Surf., B.

[cit23] Griffin W. C. (1949). Classification of surface-active agents by ‘HLB’. J. Soc. Cosmet. Chem..

[cit24] Tadros T. F. , Applications of Surfactants in Emulsion Formation and Stabilisation, in Applied Surfactants: Principles and Applications, Wiley-VM, Weinheim, 2005, pp. 115–185

[cit25] Uchegbu I. F., Vyas S. P. (1998). Non-ionic surfactant based vesicles (niosomes) in drug delivery. Int. J. Pharm..

[cit26] Hao Y., Zhao F., Li N., Yang Y., Li K. (2002). Studies on a high encapsulation of colchicine by a niosome system. Int. J. Pharm..

[cit27] Carafa M., Santucci E., Lucania G. (2002). Lidocaine-loaded non-ionic surfactant vesicles: characterization and *in vitro* permeation studies. Int. J. Pharm..

[cit28] Estupiñan O. R., Garcia-Manrique P., Blanco-Lopez M. D. C., Matos M., Gutiérrez G. (2020). Vitamin D3 Loaded Niosomes and Transfersomes Produced by Ethanol Injection Method: Identification of the Critical Preparation Step for Size Control. Foods.

[cit29] Gianasi E., Cociancich F., Uchegbu I. F., Florence A. T., Duncan R. (1997). Pharmaceutical and biological characterisation of a doxorubicin-polymer conjugate (PK1) entrapped in sorbitan monostearate Span 60 niosomes. Int. J. Pharm..

[cit30] Mehta S. K., Jindal N. (2013). Formulation of Tyloxapol niosomes for encapsulation, stabilization and dissolution of anti-tubercular drugs. Colloids Surf., B.

[cit31] Judy E., Lopus M., Kishore N. (2021). Mechanistic insights into encapsulation and release of drugs in colloidal niosomal systems: biophysical aspects. RSC Adv..

[cit32] Yeo P., Chooi Ling L., Soi Moi C., Ling A., Koh R. Y. (2018). Niosomes: A review of their structure, properties, methods of preparation, and medical applications. Asian Biomed..

[cit33] Devaraj G. N., Parakh S. R., Devraj R., Apte S. S., Rao B. R., Rambhau D. (2002). Release studies on niosomes containing fatty alcohols as bilayer stabilizers instead of cholesterol. J.
Colloid Interface Sci..

[cit34] Ohsawa T., Miura H., Harada K. (1985). Improvement of encapsulation efficiency of water-soluble drugs in liposomes formed by the freeze-thawing method. Chem. Pharm. Bull..

[cit35] MacDonald R. C., MacDonald R. I., Menco B. P., Takeshita K., Subbarao N. K., Hu L. R. (1991). Small-volume extrusion apparatus for preparation of large, unilamellar vesicles. Biochim. Biophys. Acta.

[cit36] Bangham A. D., Standish M. M., Watkins J. C. (1965). Diffusion of univalent ions across the lamellae of swollen phospholipids. J. Mol. Biol..

[cit37] Jibry N., Heenan R., Murdan S. (2004). Amphiphilogels for Drug Delivery: Formulation and Characterization. Pharm. Res..

[cit38] González-Menéndez E., Fernández L., Gutiérrez D., Pando D., Martínez B., Rodríguez A., García P. (2018). Strategies to Encapsulate the Staphylococcus aureus Bacteriophage phiIPLA-RODI. Viruses.

[cit39] Berne B. J. and Pecora R., Dynamic Light Scattering: With Applications to Chemistry, Biology, and Physics, Courier Corporation, Mineola, New York, 2000

[cit40] LS Spectrometer User's Manual, Version 5.0, LS Instruments AG, 2018

[cit41] Ježková M., Šrom O., George A. H., Kereïche S., Rohlíček J., Šoóš M. (2023). Quality assessment of niosomal suspensions. J. Colloid Interface Sci..

[cit42] Mastronarde D. N. (2005). Automated electron microscope tomography using robust prediction of specimen movements. J. Struct. Biol..

[cit43] Schindelin J., Arganda-Carreras I., Frise E. (2012). *et al.*, Fiji: an open-source platform for biological-image analysis. Nat. Methods.

[cit44] Knight C. J., Hub J. S. (2015). MemGen: a general web server for the setup of lipid membrane simulation systems. Bioinformatics.

[cit45] Vanommeslaeghe K., Hatcher E., Acharya C., Kundu S., Zhong S., Shim J., Darian E., Guvench O., Lopes P., Vorobyov I., Mackerell Jr A. D. (2010). CHARMM general force field: A force field for drug-like molecules compatible with the CHARMM all-atom additive biological force fields. J. Comput. Chem..

[cit46] Huang J., Rauscher S., Nawrocki G., Ran T., Feig M., de Groot B. L., Grubmüller H., MacKerell A. D. (2017). CHARMM36m: an improved force field for folded and intrinsically disordered proteins. Nat. Methods.

[cit47] Ong E. E. S., Liow J.-L. (2019). The temperature-dependent structure, hydrogen bonding and other related dynamic properties of the standard TIP3P and CHARMM-modified TIP3P water models. Fluid Phase Equilib..

[cit48] Jo S., Kim T., Iyer V. G., Im W. (2008). CHARMM-GUI: A web-based graphical user interface for CHARMM. J. Comput. Chem..

[cit49] Brooks B. R., Brooks III C. L., Mackerell Jr A. D. (2009). CHARMM: The biomolecular simulation program. J. Comput. Chem..

[cit50] Essmann U., Perera L., Berkowitz M. L., Darden T., Lee H., Pedersen L. G. (1995). A smooth particle mesh Ewald method. J. Chem. Phys..

[cit51] Nosé S. (1983). A molecular dynamics method for simulations in the canonical ensemble. Mol. Phys..

[cit52] Hoover W. (1985). Canonical dynamics: Equilibrium phase-space distributions. Phys. Rev. A.

[cit53] Parrinello M., Rahman A. (1981). Polymorphic transitions in single crystals: A new molecular dynamics method. J. Appl. Phys..

[cit54] Hess B. (2008). A Parallel Linear Constraint Solver for Molecular Simulation. *J. Chem. Theory Comput.*.

[cit55] Miyamoto S., Kollman P. A. (1992). Settle: An analytical version of the SHAKE and RATTLE algorithm for rigid water models. J. Comput. Chem..

[cit56] Abraham M. J., Murtola T., Schulz R., Páll S., Smith J. C., Hess B., Lindahl E. (2015). GROMACS: High performance molecular simulations through multi-level parallelism from laptops to supercomputers. SoftwareX.

[cit57] Bartelds R., Nematollahi M. H., Pols T., Stuart M. C. A., Pardakhty A., Asadikaram G., Poolman B. (2018). Niosomes, an alternative for liposomal delivery. PLoS One.

[cit58] Tang W. L., Chen W. C., Roy A., Undzys E., Li S.-D. (2016). A Simple and Improved Active Loading Method to Efficiently Encapsulate Staurosporine into Lipid-Based Nanoparticles for Enhanced Therapy of Multidrug Resistant Cancer. Pharm. Res..

[cit59] Zhao Y.-Z., Lu C.-T. (2009). Increasing the entrapment of protein-loaded liposomes with a modified freeze-thaw technique: a preliminary experimental study. Drug Dev. Ind. Pharm..

[cit60] Gonzalez Gomez A., Syed S., Marshall K., Hosseinidoust Z. (2019). Liposomal Nanovesicles for Efficient Encapsulation of Staphylococcal Antibiotics. ACS Omega.

[cit61] National Center for Biotechnology Information , PubChem Compound Summary for CID 5411, Tetracaine, https://pubchem.ncbi.nlm.nih.gov/compound/Tetracaine

[cit62] Kučerka N., Nieh M.-P., Katsaras J. (2011). Fluid phase lipid areas and bilayer thicknesses of commonly used phosphatidylcholines as a function of temperature. Biochim. Biophys. Acta.

[cit63] Ritwiset A., Maensiri S., Krongsuk S. (2024). Insight into molecular structures and dynamical properties of niosome bilayers containing melatonin molecules: a molecular dynamics simulation approach. RSC Adv..

[cit64] Najafian S., Marsusi F., Mirabbaszadeh K. (2024). Martini 3 coarse-grained models of the niosomes based on Span60 and Tween60. J. Mol. Liq..

