# Supplementary material for: SPAN 60 and TWEEN 60 surfactant-based niosomal vesicles: from preparation to molecular dynamics simulations
Source: Nanoscale Adv. 2026 Aug 3;8(21):6175–88. doi: 10.1039/d6na00065g (PMC13459183; doi:10.1039/d6na00065g)
Supplement: Supplementary file 1 [file NA-008-D6NA00065G-s001.pdf]

Supplementary Information (SI) for Nanoscale Advances.
This journal is © The Royal Society of Chemistry 2026

Supplementary Information

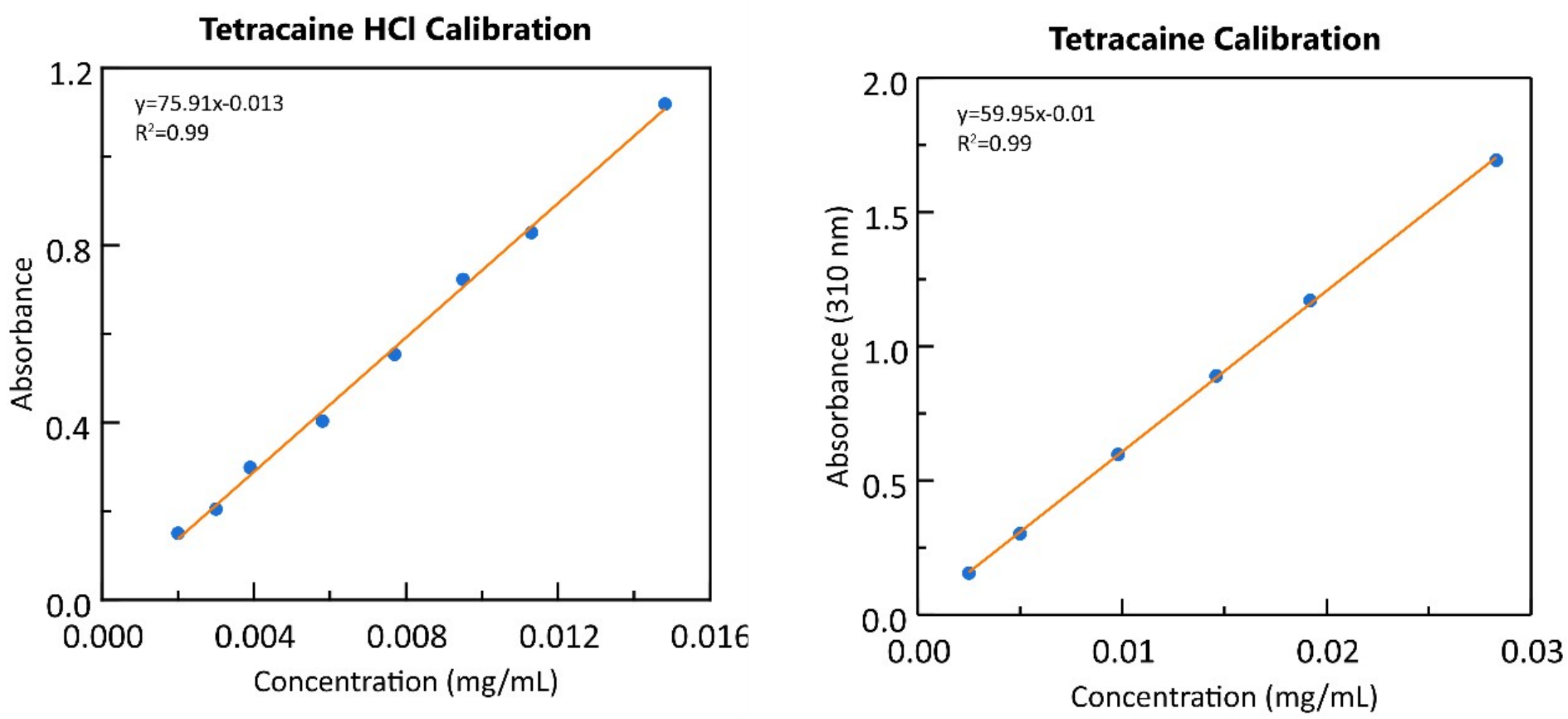

Fig S 1. Calibration curves for Tetracaine HCl and Tetracaine at their characteristic peak wavelengths (312 and 310, respectively). The solvent used was water for both systems.
